# The Meaning of Immune Reconstitution after Alemtuzumab Therapy in Multiple Sclerosis

**DOI:** 10.3390/cells9061396

**Published:** 2020-06-03

**Authors:** Simona Rolla, Alessandro Maglione, Stefania Federica De Mercanti, Marinella Clerico

**Affiliations:** Department of Clinical and Biological Sciences, University of Turin, 10043 Orbassano (TO), Italy; alessandro.maglione@unito.it (A.M.); stefania.demercanti@unito.it (S.F.D.M.); marinella.clerico@unito.it (M.C.)

**Keywords:** Alemtuzumab, multiple sclerosis, mode of action, immune reconstitution

## Abstract

Alemtuzumab is a monoclonal antibody that binds to CD52, a protein present on the surface of mature lymphocytes, but not on the stem cells from which these lymphocytes are derived. It is currently used as an immune reconstitution therapy in patients with relapsing–remitting multiple sclerosis. Alemtuzumab treatment is an intermittent infusion that induces long-term remission of Multiple Sclerosis also in the treatment-free period. After the robust T and B cell depletion induced by alemtuzumab, the immune system undergoes radical changes during its reconstitution. In this review, we will discuss the current knowledge on the reconstitution of the lymphocyte repertoire after alemtuzumab treatment and how it could affect the development of side effects, which led to its temporary suspension by the European Medical Agency.

## 1. Introduction

Multiple sclerosis (MS) is a chronic inflammatory demyelinating disease of the central nervous system (CNS) that leads to demyelination and axonal damage following the activation of both innate and adaptive immune system [1]. The experimental evidence coming from the murine model of MS, the Experimental Autoimmune Encephalomyelitis (EAE) and biological samples of MS patients, give us the actual comprehension of the immunological processes underlying the immunopathogenesis of MS. An imbalance in both T and B cells immune regulatory network is at the basis of the autoreactive immune response and is influenced by genetics and environmental factors. Among T cells, Th17 cells can efficiently cross the blood–brain barrier, promote its disruption and induce the activation of other inflammatory cells in the CNS [2]; CD8^+^ T cells can mediate damage to resident cells and axons potentially by the recognition of CNS derived peptides [3]. By contrast, T regulatory (Treg) cells that normally control inflammation are impaired in number and function [4] and allow autoreactive T cells to induce CNS damage. B cells contribute to the disease via both antibody-dependent and -independent mechanisms, which are essential for antigen presentation and co-stimulation of T cells, for the production of cytokines and to produce antibodies that will target components of the CNS [5]. Besides the adaptive immune response, microglial cells produce, in the CNS, pro-inflammatory cytokines and reactive oxygen and nitrogen species that contribute to neuroinflammation and destruction of neurons [6].

This complex cellular and molecular network that drives MS disease suggests that the preferred therapy for MS should be targeting multiple components. Disease-modifying therapies (DMTs) can reduce the frequency and severity of MS relapse and slow disease progression by modulating the immune system [7,8]. There are currently several drugs approved by the Food and Drug Administration (FDA) for modifying MS; they come as injectables, infusions and oral treatments. Immune reconstitution therapy (IRT) is an emerging concept for the treatment of MS [9,10]. The aim of IRTs is to eliminate a pathogenic immune repertoire through intense short-term immunosuppression, and to subsequently rebuild a new and healthy immune system with the goal to re-establish a persistent immune tolerance [11]. After a period of deep immune depletion, the immune system undergoes reconstitution and radical changes in the lymphocyte repertoire and regains its ability to respond to infections. IRTs include autologous hematopoietic stem cell transplantation (AHSCT), alemtuzumab, cladribine tablets and anti-CD20 agents. The most extensively studied IRT is alemtuzumab and here we will recapitulate the current understanding of its long-term efficacy and common adverse events, through an immunological point of view.

## 2. Alemtuzumab: From Bench to Bedside

### 2.1. CD52 Structure and Function (Alemtuzumab Mechanism of Action)

**Figure 1:**
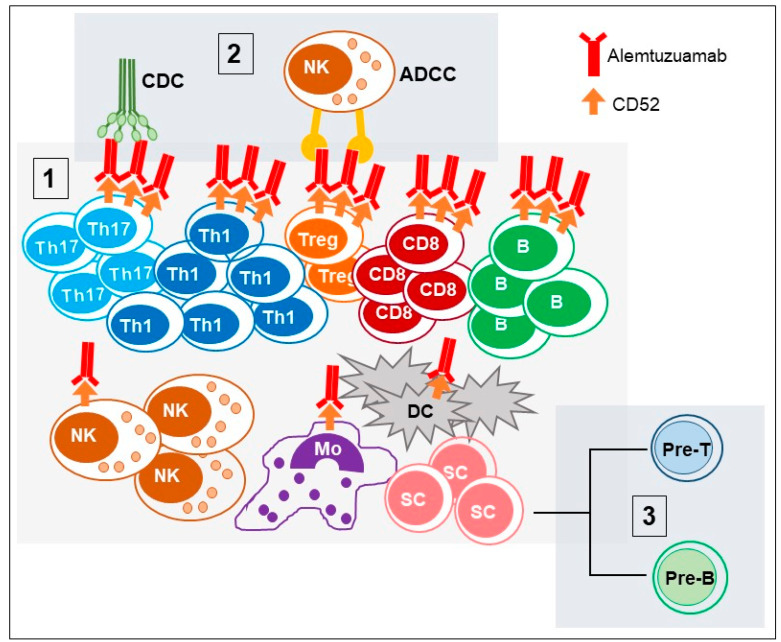
Alemtuzumab mechanism of action. Alemtuzumab exerts its function through three main phases: 1. Selection: Alemtuzumab selectively binds to CD52 antigen that is highly expressed on T (here showed as the main subtypes involved in MS: Th17, Th1, Treg and CD8^+^ cells) and B cells and at low level on NK cells and macrophages (Mo) and peripheral DCs. 2. Depletion: Alemtuzumab induce depletion of T and B cells through complement mediated cytotoxicity (CDC) and antibody dependent cellular cytotoxicity (ADCC). 3. Repopulation: New T and B cells originate from stem cells (SC) that escape alemtuzumab depletion, as they do not express the CD52 antigen) or by homeostatic proliferation of lymphocytes that escape depletion.

Alemtuzumab is a recombinant humanized immunoglobulin G1 (IgG1) monoclonal antibody directed against the CD52 surface antigen, a small (12 amino acids) glycosylphosphatidylinositol (GPI)-anchored protein of undefined function [12]. CD52 is expressed on the leukocyte membrane during the differentiation process while it is absent on the membranes of hematopoietic precursors. In humans, CD52 is expressed at high levels in T and B lymphocytes and at lower levels in natural killer (NK) cells, monocytes, macrophages, eosinophils and monocyte-derived peripheral blood dendritic cells (DC) [13], while it is absent (or expressed at very low levels) in tissue resident DCs [14], neutrophils and hematopoietic stem cells [15] (Figure 1).

Even when under investigation, the biological functions of CD52 could include cell adhesion and lymphocyte proliferation and may be involved in the co-stimulation of T lymphocytes, as well as in their migration [16]. Interestingly, activated CD4+ T cells and expressing high levels of CD52 could have regulatory activities on T effector lymphocytes [17,18]. These CD4^+^CD25^+^CD52^high^ suppressive T cells, which are different from classical CD4^+^CD25^high^FOXP3^+^ Treg cells, exert their function through the release of CD52 soluble molecules by phospholipase-C. The target of this soluble CD52 is the inhibitory receptor sialic acid-binding immunoglobulin-like lectins-10 (Siglec-10), thus impairing the phosphorylation of the T cell receptor (TCR)-associated kinases Lck and Zap70 and thus T cell activation. However, these CD4^+^CD25^+^CD52^high^ have never been reported in MS patients and seems not to be the main mode of action of alemtuzumab. A few minutes after infusion, alemtuzumab leads to depletion of CD52 positive cells through antibody-dependent cell-mediated cytolysis (ADCC) and complement-dependent cytolysis (CDC), and induces caspase-dependent apoptosis (Figure 1). In particular, the recruitment of the attack complex factors (C5b, C6, C7, C8 and then numerous C9 molecules), which create pores in the cell membrane and cause cell death by complement-mediated cytolysis, plays the greatest part in humans [19].

### 2.2. Alemtuzumab for the Treatment of MS: Preclinical Studies in Transgenic Mice and EAE Models

In the preclinical phase, the mechanism of action of alemtuzumab on lymphocytes was demonstrated in transgenic mice expressing human CD52 (huCD52) molecule due to the inability of alemtuzumab to cross react with mouse CD52 [15]. HuCD52 mice were able to mount normal immune responses and replay the tissue distribution and expression patterns of the human immune compartment. A single injection of alemtuzumab recapitulated changes observed in human studies in serum cytokine levels and depletion of peripheral blood T and B lymphocytes, with a lower effect on neutrophils and NK cells, and no damage in the hematopoietic or progenitor stem cells [20,21]. Lymphocyte depletion occurs mainly in the peripheral circulation, while the reduction in the number of lymphocytes in the lymphoid organs (spleen, lymph nodes, bone marrow and thymus) is marginal [15]. Moreover, lymphocyte repopulation was very similar to that observed in humans: B cells returned to baseline levels seven to ten weeks post alemtuzumab, whereas T cells recovered more slowly, reaching baseline levels only by 25 weeks. A possible explanation for the differential timing of T and B cell repopulation in the blood could be related to the fact that alemtuzumab did not significantly affect the bone marrow, thus allowing for the rapid recovery of B cells; by contrast, it induces a partial depletion of single-positive and double-positive thymocytes, which could explain the slower recovery of T cells. Frequencies of CD4^+^CD25highFoxP3^+^ cells were found to increase, even though expressing equivalent levels of surface CD52 compared to all CD4^+^ T cells and their expansion could be connected to the resistance of EAE mice to relapses. Moreover, recent findings show that also CD8 double-negative (DN) T cells are hyper-repopulated after alemtuzumab treatment in EAE mice. DN T cells have frequently been associated with suppression of immune responses, highlighting another possible mechanism to suppress autoimmunity beside the action of Treg cells [22].

The efficacy of anti-CD52 therapy was also assessed in the EAE models, which allow to demonstrate both the immunosuppressive action of anti-CD52 antibody and parallel the long term ability of alemtuzumab to control disease in Relapsing Remitting MS (RRMS) patients [23]: Anti-murine CD52 antibody was able to reduce the number of circulating and CNS infiltrating antigen-specific T cells; EAE mice treated at early stages of the disease maintained stable disease up to 90 days without retreatment despite complete lymphocyte repopulation in the blood and lymphoid organs. In addition to induced peripheral lymphopenia, some effects have been observed in CNS. Recently, it has been shown that alemtuzumab treatment almost completely depleted CNS infiltrates and B cell aggregates in the CNS of mice with myelin basic protein (MBP)-proteolipid protein (PLP)-induced EAE [24]. While the neuroprotective effects of alemtuzumab have been suggested, anti-CD52 treatment does not appear to affect microglia functions in EAE [25]. Overall, data from murine studies suggested that the beneficial effects of alemtuzumab in MS appear to be mediated exclusively by peripheral immune mechanisms.

### 2.3. Clinical Studies of Alemtuzumab in MS

Alemtuzumab was initially approved for the treatment of B-cell chronic lymphocytic leukemia in people who have been treated with alkylating agents and who have failed fludarabine therapy and in bone marrow transplanted patients, given its pronounced cytolytic effects [26].

In 1991 the first MS patients at Addenbrooke’s Hospital in the United Kingdom (UK) were treated with alemtuzumab. These early investigations revealed alemtuzumab’s high efficiency in ameliorating inflammatory responses in relapsing–remitting as well as in Secondary Progressive MS (SPMS) [27]. The use of alemtuzumab, commercially known as Lemtrada^®^, for the therapy of RRMS, was approved by the European Medicines Agency (EMA) on September 12, 2013 (EMEA/H/C/003718, MA number: EU/1/13/869), at a dosage of 12 mg/day administered intravenously for five consecutive days at (60 mg total dose) and three consecutive days after 12 months (36 mg total dose). In November 2014, alemtuzumab was also approved by the FDA.

In MS patients, the therapeutic cycle of alemtuzumab induces immediate lymphopenia that lasts for a few years, although the average half-life of alemtuzumab is six days after the last dose [28] and it has no effects on the lymphocytes located in the primary and secondary lymphatic organs [15]. Several clinical trials have confirmed the clinical efficacy of alemtuzumab in RRMS with high disease activity [29,30,31]. The international multi-center Phase II clinical study called Campath-1H in Multiple Sclerosis (CAMMS223) compared low and high doses of alemtuzumab and IFNβ-1a in patients with RRMS with high disease activity and who were previously untreated. In this study, alemtuzumab was more effective in terms of both disability and relapse than IFNβ-1. The 223 patients undergoing alemtuzumab therapy received a low dose (12 mg/day) or a high dose (24 mg/day) of drug administered intravenous (i.v.) for five consecutive days at the baseline and three consecutive days at 12 and 24 months; the 111 patients treated with IFNβ-1a received a dose of 44 µg subcutaneous (s.c.) three times a week [29]. The results of the CAMMS223 study were subsequently confirmed by phase III clinical studies, called Comparison of Alemtuzumab and Rebif^®^ Efficacy in Multiple Sclerosis, CARE-MS I and CARE-MS II, aimed at exploring the efficacy of alemtuzumab in cohorts with broader inclusion criteria. In the CARE-MS I study (or CAMMS323) [30], patients aged between 18 and 50 with RRMS who received no previous treatment (except for steroids) were enrolled with a baseline Expanded Disability Status Scale (EDSS) of less than or equal to 3.0 and appearance of disease symptoms in the previous five years. In the CARE-MS II study (or CAMMS324) [31] patients aged between 18 and 50 years, with a baseline EDSS of less than or equal to 5.0 and symptoms occurring in the previous 10 years, who had developed at least one relapse after previous treatment with IFN-β or glatiramer acetate carried out for at least six months were enrolled. Patients were randomized 2:1 to receive two annual treatment cycles with alemtuzumab (12 mg/day i.v.) once daily for five consecutive days at baseline and 3 consecutive days at 12 months) or with IFNβ-1a at high dose (Rebif^®^, 44 μg s.c. three times a week). From this phase III study it emerged that alemtuzumab can be used in patients with RRMS refractory to treatment with first-line drugs to reduce the risk of recurrence and the sustained accumulation of disability [31].

In 2017, Havrdova et al. published a report of the interim results through three years of an extension study in patients treated with alemtuzumab during the CARE-MS I trial, with a total of five years of follow-up from CARE-MS I enrollment [32]. In this extension study 68.5% patients did not receive additional alemtuzumab treatment. The annual relapse rate (ARR) and the brain volume loss remained significantly low through the years most patients (61.7%, 60.2% and 62.4%) had NEDA in years 3, 4 and 5. In conclusion, based on these data, alemtuzumab showed durable efficacy through five years in the absence of continuous treatment, with most patients not receiving additional courses [32]. In the alemtuzumab CARE-MS II five-year follow-up study [33] 59.8% of patient received no alemtuzumab retreatment. ARR was low in each extension year (years 3–5: 0.22, 0.23, 0.18). In years 3, 4 and 5, proportions of patients with NEDA were 52.9%, 54.2% and 58.2%, respectively. Moreover, the brain volume loss remained low in the extension phase [33]. The results show durable efficacy through five years in the absence of continuous treatment [33]. A 2019 study [34] evaluated the six-year outcomes in patients who relapsed between alemtuzumab courses 1 and 2, i.e., the “early relapsers”. The results show the outcome of the “early relapsers” improved after completing the second alemtuzumab course, supporting the fact that administering the approved two-course regimen should maximize the clinical benefit [34]. A 2019 study [35] shows that quality of life of patients treated with alemtuzumab ameliorates over a six year post- alemtuzumab treatment period, regardless the independently the occurrence of autoimmune thyroid adverse events.

## 3. Immune System Reconstitution after Alemtuzumab Treatment

The therapeutic efficacy of alemtuzumab has been associated not only with induced lymphopenia but also with the peculiar modality of alemtuzumab-induced repopulation of immune cells that results in reduced relapses and delay of disease progression. After depletion of T and B lymphocytes from circulation, the remaining lymphocytes retain their normal function in terms of the ability to respond to new pathogenic stimuli and immunological memory to recall antigens [36].

Even if the rate differs among the subsets, as better described below, the speed of lymphocytes repopulation is unaltered between the subjects treated with alemtuzumab who develop relapses and those who do not develop them. This suggests that the nature of immune system repopulation is more important than the number of lymphocytes itself, in particular T and B cell subsets [37]. Several studies, especially phase II and III trials, have addressed how repopulation of CD4^+^, CD8^+^ T and CD19^+^ B cells occurred in alemtuzumab treated MS patients [38,39], but only a few focused on the evaluation of their subsets and cytokines produced as their level has been shown to be related to therapy response [40,41,42,43,44,45], thus contributing to clarifying the long-lasting efficacy of alemtuzumab. Here we will review the current knowledge on immune reconstitution after alemtuzumab analyzing together the various studies and trying to outline a common mechanism (summarized in Table 1).

### 3.1. Reconstitution of T Cells Subsets

Alemtuzumab rapidly and drastically depletes the number of circulating CD4^+^ and CD8^+^ T cells after each treatment cycle (by ≥95% and about 85% of baseline, respectively, at one month) [30,31,46]. Then, CD4^+^ and CD8^+^ cell numbers return to their lowest normal limit with different kinetics depending on the studies (reviewed in Sellner et al., [9]); overall these studies indicate that CD4^+^ T cells are very slow to repopulate, whereas CD8^+^ recovered earlier with a median time of 32 and 19.5 months, respectively [37]. In line with these results on absolute counts, we show that alemtuzumab administration strongly affected the percentage of CD4^+^ cells: It was reduced by 66% at month 6, by 51% at month 12 and then slowly increased returning to baseline around month 48 from the first administration course [47,48]. The percentage of CD8^+^ T cells in lymphocytes returned to baseline at 15 months [39]. Data coming from CARE-MS I and II trials and analyzed by Baker et al. [49] showed that both naïve (CD45RA^+^) and memory (CD45RA^−^) CD4 and CD8 T cells were depleted within 1 month after each administration course, but their reconstitution is different: Memory CD4^+^ T cells repopulate faster than naïve. T lymphocyte repopulation would seem to occur through the proliferation of mature lymphocytes that escape depletion (the so-called “homeostatic proliferation”), rather than through the proliferation of new thymic precursors [50]: T cell transfer experiments with lymphopenic animals have demonstrated that homeostatic proliferation is dependent on TCR-self-reactive stimulation [51,52] and IL-7, whose levels increase after alemtuzumab administration [39]. Because of this altered immune physiology, T-cell expansion under these circumstances may favor the proliferation of chronically activated oligoclonal memory T cells favoring the development of secondary autoimmune conditions that are frequently observed as an adverse event of alemtuzumab treatment [49,53]. However, over time, thymic T cell recovery gains more importance as demonstrated by increasing clonal diversity [50].

Looking at the different CD4^+^ T cell subsets, a relative expansion of CD4^+^CD25^+^CD127^low^ Treg cells and Th2 cells, coupled with a preferential reduction of Th17 cells, rather than Th1 cells observed during immune reconstitution [39,47,54]. These changes underline the efficacy of alemtuzumab in modulating the adaptive immune system toward an immunotolerant environment. In detail, we observed that the number of pathogenic Th17 and Th1 cells was decreased until month 48 [47,55], even if their percentage of CD4^+^ T cells fraction was unchanged during the overall follow up. However, another study shows a significant decrease in Th17 and Th1 percentage at months 12 and 24 [39]. In line with these results, circulating the number of cytokines and chemokines related to Th17 (IL-1β, IL-6, IL-17A, IL-17F, IL-22, IL-23, IL-26, TNF-α, CCL20) and Th1 (IL-12, IFN-γ, CXCL10) cells were found decreased [39,47,55]. Th2 cells producing IL-4, IL-10 and TGF-β1 were found to increase starting from month 3 [39].

Of particular meaning are the results on Treg cells. CD4^+^CD25^+^CD127low Treg cells were depleted by 81–86% after alemtuzumab infusion; during the reconstitution phase of the circulating lymphocyte repertoire, Treg cells preferentially expanded within the CD4^+^ lymphocyte population, reaching peak expansion at month 1 [39] and becoming significantly higher compared to baseline at month 24, where a restored suppressive function was also observed [47]. After alemtuzumab, Treg cells were able to suppress both syngeneic conventional T-cells [56] and antigen-specific autoreactive Th17 and Th1 cells [47,57]. Accordingly, FoxP3 expression and cytokines related to Treg cells (TGF-b and IL-10) increased. As for total CD4^+^ T cells, memory Treg cells were preferentially (89% of Treg cells) expanded rather than the naïve ones [47] as a result of homeostatic proliferation induced by IL-7, rather than thymopoiesis, of Treg cells resistant to alemtuzumab depletion. However, why these Treg cells become more suppressive during the repopulation period needs to be further investigated as it is not clear if it is the result of enhanced cytokine production by Treg cells themselves, rather than an altered composition and reactivity of repopulated CD4^+^ T cells that are more susceptible to regulation.

### 3.2. B Cells

The B cell population is affected by alemtuzumab treatment too, but its recovery, both in counts and in subtypes, strictly differs from T cells. B cell numbers in the circulation were low at month 12 and returned to normal levels at month 24. Although in the first weeks after administration of alemtuzumab, the mature CD19^+^ naïve B lymphocytes decreased (<85%), a hyper-repopulation of immature B cell clones (to 160–180% of baseline levels) was observed at 3–6 months [49]. These immature B cells converted into mature B cells (identified as transitional B cells) within 12 months and dominated the B cell pool [9,49]. Memory B cell counts remained low throughout the 12–48-month post-treatment periods. These changes in B cells have been coupled with serum alterations of the B-cell activating factor (BAFF), an essential factor for the survival and differentiation of B lymphocytes [58]. The peculiar reconstitution of the B-cell compartment has been suggested to be at the base of the development of secondary autoimmunity that was frequently observed in alemtuzumab treated patients.

As for Treg cells, also the fraction of Breg cells increase post alemtuzumab [57]: They significantly increased five months after the first course and remained elevated until 11 months after the second course. The reconstitution of Breg cells involve both highly expressing programmed death ligand-1 B cells (CD19^+^PD-L1^hi^ cells) that exert regulatory function through cell-to-cell contact via interaction of CD19^+^PD-L1^hi^ cells with PD-1 on T cells and both the immature transitional B cell subset (CD19^+^CD24^hi^CD38^hi^) that produce IL-10. In particular, a deficiency of CD19^+^CD24^hi^CD38^hi^ B cell subset has been shown cells during relapse compared to remission and healthy subjects. Following alemtuzumab, the distribution of B cells shifts towards naïve phenotype and this Breg deficiency is restored highlighting the possible mechanism of protection related to Breg cells [59].

Despite induced B lymphopenia, alemtuzumab appears to have no significant effect on immune responses to vaccines [36]. RRMS patients treated with alemtuzumab maintain humoral immunological memory and the ability to develop an immune response against various vaccines: anti-diphtheria, tetanus and poliomyelitis, type b anti-Haemophilus influenzae, conjugate vaccine anti-meningococcus C and anti-pneumococcal polysaccharide [58].

### 3.3. DC/Neutrophils/Macrophages/NK Cells

The alemtuzumab treatment also highlighted transient effects on the components of innate immunity (e.g., neutrophils, macrophages, NK cells). As compared to CD4^+^ T cells, innate myeloid and lymphoid cells from MS patients express less CD52 on their cell surface, so, the count of these cells is affected minimally or transiently after each course of treatment [60]. Neutropenia is generally mild and recovers by the end of the infusion cycle [61]. Alemtuzumab treatment did not strongly affect dendritic cells (DC); however, Gross and colleagues found reduced number of circulating plasmacytoid-DC (a particular subset of DC able to elicit pro-inflammatory immune response) in the 6th month of alemtuzumab treatment compared to baseline, although the production of GM-CSF and IL-23 in these cells remained unchanged [62]. NK cells are reduced to a lesser extent than T and B lymphocytes, which may relate to their lower expression of CD52 antigen [63]. Interestingly, a recent study shows that the subset of CD56^bright^ NK cells expands, although their cytolytic activity is unaffected. It has been proposed that this remodeling of the innate immune system could have potential immunoregulatory properties in MS, thus contributing to the long-term efficacy of alemtuzumab and to preserve MS patients immunocompetence [62].

Altogether, these studies suggest that the immunomodulatory effects of alemtuzumab occur through lymphocyte depletion and repopulation [36,38,39,47,49,54,58,60,61,62,63,64,65,66] and include an increased presence of Treg cells, an increased presence of memory T and B cells and transient effects on the components of innate immunity (e.g., neutrophils, macrophages, NK cells). These mechanisms underlie the anti-inflammatory effect of alemtuzumab and its ability in decreasing relapses, thus delaying the progression of the disease, but also suggests some interesting connection with the development of secondary autoimmune conditions and other adverse effects.

## 4. Beyond Depletion and Immune Reconstitution: Alemtuzumab Management

Cellular and molecular changes of the immune system after alemtuzumab administration could be at the basis of the development of adverse effects:

- within 2–6 h after alemtuzumab infusion the so called “cytokine-release syndrome” was observed: The participation of monocytes, macrophages and NK cells to the lysis of lymphocytes mediated by alemtuzumab results in an acute induction of several pro-inflammatory cytokines, such as TNF-α, IL-6 and IFN-γ;
- the depletion of CD8^+^ could be associated with the increased risk of viral infection;
- the recovery of T cells, as results from homeostatic proliferation rather than thymic reconstitution could be associated with the development of secondary autoimmunity;
- the hyper population of naïve B cells could be responsible for the secondary B cell autoimmunity.

### 4.1. Development of Secondary Autoimmunity

Alemtuzumab is associated with high incidence of secondary autoimmunity that could limit its usage. Baker and colleagues proposed that the B-cell depletion followed by hyperpopulation of B cells, in a phase with lower T cell regulation, as well as the hyperpopulation of naïve B cells in association with long-lasting depletion of memory B cells could be the key factors in the development of autoimmunity in an individual with genetic susceptibility for autoimmunity [49]. Moreover, the recovery of T cells comes from a peripheral expansion and could promote immune cell population that respond to self [9]. Autoimmunity occurs generally 16 months after the last alemtuzumab administration [67], reaching the peak in the third year after starting alemtuzumab treatment [33]: The development of autoantibodies occurs months to years after alemtuzumab administration because it requires CD4^+^ T-cell involvement, which regenerate only six months to three years after depletion, with a subsequent delay between B cell hyperreactivity and development of autoimmunity [49]. Another discussed risk factor that may predispose to autoimmunity is an overproduction of IL-21. IL-21 may drive cycles of T cell expansion and apoptosis to excess increasing the stochastic opportunities for T cells to encounter self-antigen and, hence, lead to autoimmunity [53,68]. On the other hand, IL-21 influences B-cell function: IL-21 signaling in B cells is required, together with CD4^−^ T cell cooperation, for their differentiation to antibody-producing plasma cells [69], that in turn could be important in the development of antibody-induced autoimmunity [70].

The organ most involved in autoimmunity is the thyroid, with most analysis reporting an occurrence in 17% to 34% of patients [71]. Graves’ disease, occurring in 60% to 70% of cases, is the main cause of thyroid disfunction [29,67]. The individual risk is threefold higher in smokers and sevenfold greater in patients wo have a family history, there are non-conclusive certainties regarding the role of sex as risk factor [72]. A 2018 study [71] recorded fluctuating thyroid status in Graves’ disease induced by alemtuzumab and an unexpectedly high frequency of TRAb-positive hypothyroidism, which led to the hypothesis of changing activity of anti TSH receptor antibodies (TRAb) in this context; the authors registered the existence of both blocking and stimulating TRAb in these groups of patients. Other autoimmune conditions potentially more severe encompass immune thrombocytopenic purpura (ITP) in 3% of cases and anti-Glomerular Basement Membrane disease (GBM) at much lower frequency [73].

Understanding the factors that predispose patients to this adverse effect would be crucial in guiding the clinical prescription of alemtuzumab in the long term.

### 4.2. Infection Risk

Due to the deep and long-lasting immune suppression, there are concerns regarding the risk of opportunistic infections. Considering the timing of the immunosuppression and the subsequent immune reconstitution, a higher rate of infections is to be expected principally in the first six months after dosing, with the highest risk during the first month of treatment, reducing thereafter [46,74]. Recently, analysis of six year-pooled data from trials showed that infection risk peaked after the first course of alemtuzumab and then declined over time [75]. Otherwise, serious infections are much less frequent that those observed in patients with HIV infection or other comparable CD4^+^ cells counts [29,30,46]. This phenomena could be explained by alemtuzumab mode of action that, overall, contributes to immune competence in alemtuzumab-treated patients: Innate immune cells are only marginally depleted [15]; lymphatic and tissue-resident effector T cell populations are not depleted [76] and, when re-circulating, maintain their ability to respond to chemotactic stimuli and reach inflamed tissues [77]; repopulation of T and B cells starts within a few weeks after injection [49]; finally serum titer of IgG to common viruses are unchanged by alemtuzumab treatment [73].

The most common opportunistic infections reported in clinical trials are upper and lower respiratory tract infections (nasopharyngitis, sinusitis, flu, bronchitis, pneumonia), masticatory and digestive tract infections (oral herpes, dental infections, gastroenteritis, appendicitis), infections of the urinary tract and superficial fungal infections (especially oral and vaginal candidiasis) [78]. Other documented severe infections are Pneumocystis [79], nocardiosis [80,81] and Listeria monocytogenes meningitis [82,83,84]. Moreover, active and latent tuberculosis cases have been reported [85].

Depletion of CD8^+^ T cells could be associated with the increased risk of viral infection: Infections due to Herpes Simplex Virus (HSV) and Varicella–Zoster Virus (VZV) have been frequently observed [85]. Incidences of severe herpes virus infections led to the implementation of testing for antibody to VZV before starting alemtuzumab treatment and to consider the vaccination of antibody-negative patients, as well as a prophylactic anti-herpes virus treatment with acyclovir during the first month after alemtuzumab infusion [86]. An increased risk of human papillomavirus cervicitis (HPV) [78] and occasional cases of cytomegalovirus (CMV) disease have been described [79,87,88,89]. The prognostic characteristics and risk factors for opportunistic infections in MS patients treated with alemtuzumab have yet to be accurately assessed. Currently, the recommendations are limited to anti-herpetic prophylaxis, Listeria-free diet, tuberculosis prophylaxis and annual Papillomavirus screening. Given the non-negligible risk of unforeseen infectious events, it is recommended to take into account the history of infectious diseases in patients and their status of vaccination. Moreover, it is worth considering additional prophylactic strategies, including screening for Toxoplasma gondii, viral hepatitis and preventive approaches to avoid CMV reactivation and pneumocystosis [78]. Indeed, the routine HSV prophylaxis is not protective against CMV [66]. Patients who experience constitutional symptoms or a syndrome similar to mononucleosis should be tested for CMV, with the specific PCR test for CMV reactivation. In case of confirmed CMV disease, oral aciclovir should be discontinued and treatment with ganciclovir or valganciclovir should begin [87].

### 4.3. Cardiovascolar Risk and New Worning

In 2018, the FDA issued a safety announcement warning about 13 cases of ischemic and hemorrhagic stroke and cervicocephalic arterial dissection in MS patients under alemtuzumab treatment. Twelve of the 13 cases occurred within one day and the last one within three days after alemtuzumab administration, leading in some cases to permanent disability and even death [90]. Further, five cases of spontaneous intracranial hemorrhage have been retrospectively identified from four US MS centers in correspondence published online in February 2019 [91]. Since 2019, following the occurrence of these adverse events, the Pharmacovigilance Risk Assessment Committee (PRAC) of the European Medicines Agency (EMA) has launched a review of the use of alemtuzumab in the treatment of MS. While the review is ongoing, alemtuzumab should only be started in adults with highly active relapsing–remitting multiple sclerosis despite treatment with at least two DMTs or where other therapies cannot be used. The treatment with alemtuzumab should not be discontinued if already started [90]. Healthcare professionals should consider stopping treatment in patients who develop signs of possible cerebrovascular disease and patients should immediately seek medical help if they experience symptoms [90].

Alemtuzumab treatment induces cytolysis of immune cells and a rapid reaction of inflammatory activity; the subsequent release of cytokines determines an infusion-related reaction, called cytokine release syndrome (CRS), which is a frequent adverse event of alemtuzumab treatment. The CRS is a non-antigen-specific toxicity reaction that occurs as a result of high-level immune activation [92]; CRS that occurs within 2–6 h after alemtuzumab administration clinically manifests when a large number of lymphocytes and/or myeloid cells become activated and release inflammatory cytokines [92]. The participation of monocytes, macrophages and NK cells to the lysis of lymphocytes mediated by alemtuzumab results in an acute induction of several pro-inflammatory cytokines, such as TNF-α, IL-6 and IFN-γ [93]. Serious cardiovascular and thrombotic adverse reactions that have been recently described are connected with these events. These events have been associated with a significant increase in the level of D-dimer since the first administration of alemtuzumab, indicating the activation of coagulation as a potential risk of thrombotic complications in alemtuzumab therapy [94,95]. Prophylactic pretreatment of low molecular weight heparin could be considered in patients receiving alemtuzumab.

### 4.4. Anti-Alemtuzumab Antibodies

Monoclonal antibody therapies have the potential to cause immunogenic reactions and generate anti-drug antibodies (ADA) that bind to the drug and reduce its therapeutic efficacy with a cumulative effect with repeated doses. As a result, patients during treatment may not receive the expected benefit. Following two courses of alemtuzumab treatment, ADA can be detected in blood of patients [96] most likely due to the homeostatic expansion of B cells that escape depletion (Table 1) [97]; these are binding antibodies in about 85% of patients and most of them, approximately 92%, neutralizing antibodies [96]. Failure of lymphocyte depletion has been reported in patients treated with alemtuzumab, who tested positive for neutralizing antibodies [49,98]. These results appear to be conflicting with a model-based evaluation of the effects of ADA on the pharmacokinetics and pharmacodynamics of alemtuzumab that has been conducted using data from CAMMS223, CARE-MSMS I and CARE-MSMS II studies [63]. The results of this analysis indicated that most patients are positive in the presence of ADA and ADA inhibitors. A higher percentage of patients tested positive in the second course of treatment compared to the first [63]. However, the presence of ADA (including ADA inhibitor) does not appear to have any effects on the clinical efficacy or safety of alemtuzumab, including the incidence of adverse events (AE) and infusion-associated reactions (IAR), during the two cycles of treatment [63]. Furthermore, ADA or ADA inhibitor did not appear to affect the depletion or repopulation of T or B lymphocytes and their subgroups but has shown slight effects on the depletion of NK cells. However, the depletion of NK cells is usually transient regardless of the state of the antibodies, not making this observation irrelevant from clinical efficacy [63].

High-titer neutralizing ADA responses were associated with a lack of clinical response [98,99] and, recently, Baker and coll. proposed that measurement of ADA should become routine to help physicians in the decision of re-treatment for a third or fourth alemtuzumab cycle or drug-switching [97].

**Table 1:** Effects of alemtuzumab on different immune cell types and their correlation with immune reconstitution and the generation of adverse events.

Cell Type	Alemtuzumab Effect	Involvement in Adverse Events
CD4^+^ T cells	95% depleted in 1 month. Recover in ~32 months [30,31,37,46] Memory CD4^+^ T cells repopulate faster than naïve CD4^+^ T cells [49]	Development of secondary autoimmunity conditions was related to “homeostatic proliferation” [49,53] Overproduction of IL-21 by CD4^+^ T cells could predispose to autoimmunity [53,68,70]
Treg cells	CD4^+^CD25^+^CD127^low^ Treg cells depleted by 81–86% after infusion [39] Repopulation and restored suppressive function after 24 months [47,55,56,57] 89% of restored Treg cells were memory Treg cellsas result of homeostatic proliferation [47]	The lower amount of Treg cells during the hyper-population of B cells could favor the generation of autoimmunity [49]
Th17 cells	Cells and cytokines related to (IL-1β, IL-6, IL-17A, IL-17F, IL-22, IL-23, IL-26, TNF-α, CCL20) are strongly decreased until month 48 [39,47,55]	-
Th1 cells	Cells and cytokines related to (IL-12, IFN-γ, CXCL10) are strongly decreased until month 48 [39,47,55]	-
Th2 cells	Cells and cytokines related to (IL-4, IL-10 and TGF-β1) increased in 3 months [39]	-
CD8^+^ T cells	85% depleted in 1 month [30,31,46]. Recover in ~15 months [39]	The depletion of CD8^+^ were associated with the increased risk of viral infections [85]
B cells	Mature CD19^+^ naïve B lymphocytes decreased (<85%) in one week [49] Hyper-repopulation of immature B cell clones at 3-6 months that convert into mature B cells within 12 months [9,49] Breg cells increase in 5 months [57]	The hyper population of naïve B cells in association with long-lasting depletion of memory B cells could induce secondary B cell autoimmunity [49] Homeostatic proliferation of B cells escaping depletion could contribute to generation of ADA [97]
NK cells	Partecipate in the ADCC [93] NK cells are reduced [63] The subset of CD56^bright^ NK cells is expanded [62]	Participate together with monocytes and macrophages to the “cytokine release syndrome” [93] that could bring to cardiovascular and thrombotic adverse reactions [92,94]
DC cells	Plasmacitoid DC cells are reduced in 6 months [62]	-

## 5. Conclusions

Alemtuzumab is a highly effective and selective approach to manage RRMS and improve long-term outcomes: Its effects are maintained up to six years, in absence of re-treatment in the majority of patients. Several studies had clarified the immunological mechanisms at the basis of these long-term effects, mechanisms involving mainly a particular immunoreconstitution of CD4^+^ T lymphocyte subsets, strengthening their role in MS disease pathology and therapy and a reshuffle of the immune system from inflammation toward suppression. Others were focused on explaining the development of adverse events, especially secondary autoimmunity that have been frequently observed in MS. The last suggested a role of the particular immune reconstitution of the B cell subset. These studies, together with the data coming from the clinical trials with anti-CD20 monoclonal antibodies, were identified as convincing evidence for a fundamental role of B lymphocytes in the pathogenesis of MS.

More recently, some studies were focused on the identification of biomarkers. In a study, the CD4^+^ T cell percentage under the lower normal limit at the beginning of alemtuzumab therapy seems to predict patient nonresponse to alemtuzumab [40] but needs to be confirmed in a large number of patients. Serum neurofilament light chain (NfL) proteins, one of the main cytoskeletal constitutes in neurons whose levels rise upon neuroaxonal damage in the blood, were proposed as markers of patient relapses as their levels increase in the blood 5 months before a clinical relapse [100]; this observation could be important in the clinical management suggesting a further course of alemtuzumab. A multicenter, explorative phase IV study [101] is ongoing and will combine clinical, radiological and immunological data in order to identify biomarkers for treatment efficacy and safety. Safety is indeed one of the major issues that brought EMA to initiate a review of alemtuzumab therapy following the report of new side effects. The usage of alemtuzumab is now restricted to patients with active relapsing–remitting multiple sclerosis despite a full and adequate course of treatment with at least two other disease-modifying therapies or to patients where all other disease-modifying therapies are contraindicated [90]. So, further studies aimed at defining markers that can predict both the clinical response and the occurrence of adverse effects are urgently needed to better assess the risk–benefit ratio for each patient.
